# A multispecialty consensus-based red flag checklist for the early recognition of ANCA-associated vasculitis

**DOI:** 10.3389/fimmu.2026.1818414

**Published:** 2026-04-23

**Authors:** Luca Quartuccio, Federico Alberici, Luca Cimino, Amato De Paulis, Matteo Gelardi, Luca Massacesi, Venerino Poletti, Carlo Salvarani, Florenzo Iannone

**Affiliations:** 1 Rheumatology Division, Department of Medicine, University of Udine – ASUFC Academic Hospital of Udine, Udine, Italy; 2 Nephrology Unit, University of Brescia – ASST Spedali Civili di Brescia, Brescia, Italy; 3 Ocular Immunology Unit, AUSL-IRCCS of Reggio Emilia, Reggio Emilia, Italy; 4 Department of Surgery, Medicine, Dentistry and Morphological Sciences, with Interest in Transplants, Oncology and Regenerative Medicine, University of Modena and Reggio Emilia, Modena, Italy; 5 Internal Medicine and Immunorheumatology Unit, AOU Federico II, Naples, Italy; 6 Unit of Otolaryngology - Department of Clinical and Experimental Medicine, University of Foggia, Foggia, Italy; 7 Department of Neurosciences, Drugs and Child Health (NEUROFARBA), University of Florence, and Department of Urgent Neurology, Careggi University Hospital, Florence, Italy; 8 Department of Medical Specialities , GB Morgagni Hospital/University of Bologna-Forli’, Bologna, Italy; 9 Department of Medical and Surgical Sciences (DIMEC), University of Bologna (I), Bologna, Italy; 10 Rheumatology Division, Azienda USL-IRCCS of Reggio Emilia and University of Modena and Reggio Emilia, Reggio Emilia, Italy; 11 Unit of Rheumatology, Department of Precision and Regenerative Medicine, Area Jonica (DiMePRe-J), University of Bari, Bari, Italy

**Keywords:** ANCA-associated vasculitis, early diagnosis, multidisciplinary approach, multisystem involvement, red flags

## Abstract

**Background:**

ANCA-associated vasculitis (AAV) is a rare multisystem autoimmune disease in which diagnostic delay remains common and contributes to organ damage. This study aimed to develop a multispecialty, consensus-based set of clinical, laboratory, and imaging red flags to facilitate early recognition across medical disciplines.

**Methods:**

A structured consensus process inspired by modified Delphi methodology and the RAND/UCLA Appropriateness Method was conducted. A multispecialty panel including rheumatologists, nephrologists, and specialists in internal medicine, otolaryngology, pulmonology, ophthalmology, and neurology evaluated a comprehensive list of AAV manifestations. All items were scored on a 0–10 relevance scale through inter-specialty voting. Following five intra-specialty Scientific Discussion Sessions to interpret scores and refine items, a second-round evaluation was performed, and a final meeting validated the definitive checklist.

**Results:**

Organ-specific manifestations without a better explanation involving the upper airways, kidneys, nervous system, lungs, and eyes were consistently rated as the most relevant red flags for early AAV recognition. High relevance was attributed to refractory ENT (Ear, Nose and Throat) disease, active urinary sediment, otherwise unexplained central or peripheral neurological deficits in young individuals, inflammatory ocular involvement, and pulmonary hemorrhagic features. Systemic symptoms showed lower relevance scores and were considered supportive rather than specific indicators. In parallel, a unified panel of first-level laboratory and instrumental investigations was defined to facilitate early multisystem assessment when AAV is suspected.

**Conclusions:**

This multispecialty consensus-based checklist provides a pragmatic tool to support early suspicion of AAV in routine clinical practice and may help reduce diagnostic delay.

## 1 Introduction

Anti-neutrophil cytoplasmic antibody (ANCA)-associated vasculitis (AAV) is a group of rare necrotizing systemic vasculitides of small- to medium-sized vessels, often accompanied, particularly in granulomatous forms, by tissue-destructive inflammatory masses (1–3). The three major entities, granulomatosis with polyangiitis (GPA), microscopic polyangiitis (MPA), and eosinophilic granulomatosis with polyangiitis (EGPA), present with heterogeneous yet overlapping clinical manifestations. This phenotypic variability contributes substantially to diagnostic delay, which remains a major determinant of irreversible organ damage, especially renal and pulmonary injury (4, 5), and related costs (6).

The rarity of the disease and its systemic involvement make recognition in routine clinical practice challenging, especially in the early stages. Patients often present first to organ-specific specialists (otolaryngologists, pulmonologists, ophthalmologists, neurologists) before reaching rheumatology or nephrology, which further prolongs the diagnostic pathway (7). Early identification is crucial, as timely initiation of appropriate immunosuppressive therapy can considerably modify the disease trajectory (8).

Although recent therapeutic advances, including B-cell–depleting regimens and selective C5a receptor inhibition, have improved overall outcomes and reduced glucocorticoid exposure (8, 9), these benefits can be optimally achieved when a disease-modifying treatment is initiated promptly (10). Therefore, efforts have increasingly focused on identifying clinical ‘red flags’ that can quickly raise suspicions of AAV. Previous initiatives have addressed selected contexts, such as EGPA-specific indicators (11) or ocular warning signs for immune-mediated diseases (12). However, no comprehensive, multidisciplinary set of red flags encompassing the full spectrum of AAV presentations has been formally validated.

To address this unmet need, the project “Red Flag in AAV” was launched to develop a multispecialty consensus-driven checklist integrating clinical, laboratory, and imaging indicators. The goal was to create a pragmatic tool capable of supporting early recognition of AAV across different medical specialties and facilitating timely referral to expert centers.

## 2 Material and methods

**Figure 1:**
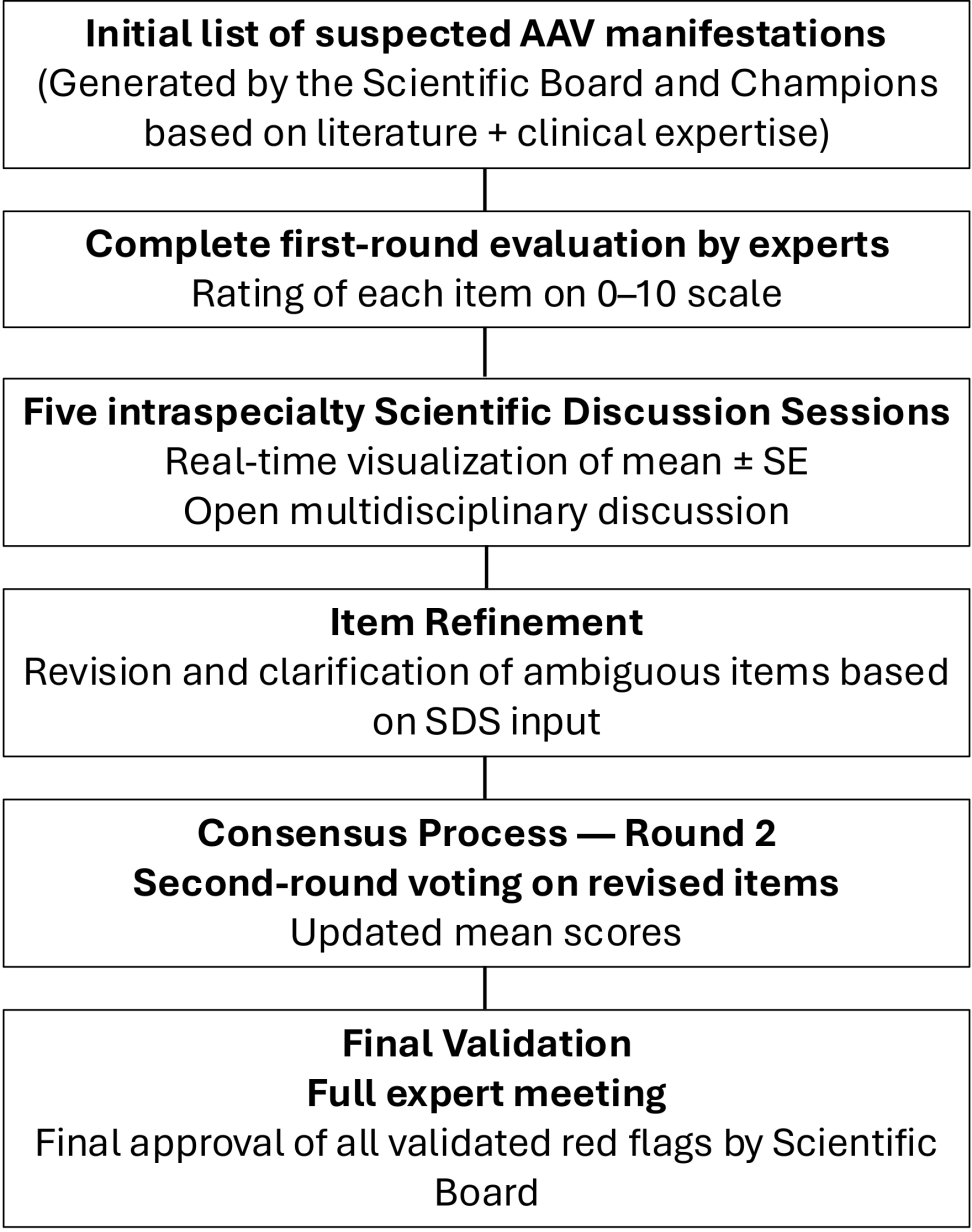
Flowchart of the study design and consensus process.

The project *“Red Flag in AAV”* was conducted through a structured multidisciplinary consensus process, designed to identify early clinical, laboratory, and imaging indicators suggestive of AAV and to translate them into a clinically usable checklist. The methodological approach followed the sequence of steps outlined in the project flowchart (**Figure 1**) and was specifically designed to address the multisystem nature and diagnostic complexity of these rare vasculitides.

The consensus process represents a hybrid model inspired by modified Delphi methodology (13) and the RAND/UCLA Appropriateness Method (RAM) (14), integrating iterative quantitative scoring with structured expert discussion.

A multispecialty panel was assembled. It included the Scientific Board, composed of three rheumatologists and one nephrologist, together with the five Clinical leads representing internal medicine, otolaryngology, pulmonology, ophthalmology, and neurology. Alongside them, 38 clinicians with documented expertise in AAV, defined by the active management of ≥20 AAV patients in follow-up within dedicated outpatient clinics and/or scientific contributions in the field of systemic vasculitis; and holding roles in national scientific societies were recruited. Their disciplinary distribution reflected the structure of the project: 8 Ear-Nose-Throat (ENT) specialists, 8 ophthalmologists, 7 pulmonologists, 8 internists, and 7 neurologists. This composition ensured that all body systems commonly involved in AAV were represented by experienced specialists, thereby supporting both the breadth and the clinical validity of the consensus process.

The process began with the generation of a preliminary list of suspected AAV manifestations (**Supplementary Table 1**). This list was drafted by the Scientific Board and the clinical leads based on current literature, accumulated clinical expertise, and everyday experience with patients exhibiting early or organ-specific features of vasculitis. The intention at this stage was to ensure that the initial item set was broad, inclusive, and representative of the full heterogeneity of AAV presentations.

All items from the preliminary list underwent a complete first-round evaluation. During this phase, all experts, irrespective of specialty, assigned a relevance score from 0 to 10 to each item. This inter-specialty evaluation was intended to capture the diagnostic perspective of different disciplines, acknowledging that early AAV manifestations frequently arise outside rheumatology settings. Scores were summarized as mean values with corresponding standard errors.

The core of the consensus process unfolded across five Scientific Discussion Sessions (SDS). These sessions were conducted within each specialty, allowing specialists to examine the scores assigned in the inter-specialty round and to discuss, from the standpoint of their clinical field, the significance of each manifestation. Each session involved real-time visualization of mean scores and their standard errors, providing a basis for structured interpretation and guided discussion. The sessions were jointly moderated by a member of the Scientific Board and the clinical lead, fostering a systematic and rigorous evaluation of each item.

Following these discussions, a dedicated refinement phase was undertaken. Items that were ambiguous, insufficiently specific, or potentially misinterpreted were reformulated to improve clarity and cross-specialty interpretability. Only manifestations previously identified by the Scientific Committee as potential red flags for AAV were considered throughout this process.

The refined items were subsequently submitted to a second-round evaluation, allowing panel members to reassess relevance after clarification and to generate updated mean scores reflecting consolidated expert opinion. Although a conventional relevance threshold was calculated for methodological transparency, no restrictive numerical cut-off was applied for item exclusion. This decision was consistent with the primary objective of maximizing diagnostic sensitivity and producing a comprehensive, clinically usable checklist.

At the end of this process, a final validation meeting was held. During this meeting, the Scientific Board reviewed the final set of scores, discussed any residual inconsistencies, and formally approved the complete set of validated red flags.

## 3 Results

All red flags and their corresponding scores derived from the SDS voting process are reported in **Table 1** and organized by clinical domain, reported symptoms, and clinical detected findings.

**Table 1:** Integrated summary of red flags voted by specialty (with mean relevance scores).

Systemic	ENT	Ophthalmology	Pulmonology	Neurology	Nephrology	Gastroenterology	Dermatology
Reported symptoms							
Persistent arthro-myalgias (6.0)	Recurrent refractory epistaxis (9.1)	Ocular pain (7.4)	Hemoptysis/blood-stained sputum (7.8)	Severe new-onset/drug-resistant headaches (7.2)		Abdominal angina (6.8)	
Fever (5.8)		Rapid-onset visual blurring with reduced acuity (7.4)	Persistent asthma (7.6)			Mucous–bloody diarrhea (6.4)	
Asthenia (5.4)		Diplopia (7.1)	Localized inspiratory and expiratory wheezing (7.1)				
Weight loss (5.4)		Red eye (6.7)					
Clinical detected findings							
	Chronic crusting hemorrhagic rhinitis (9.1)	Scleritis (9.0)	Stridor (8.8)	Mononeuritis multiplex/asymmetric polyneuropathy (9.2)	Kidney impairment with active urinary sediment (proteinuria, microscopic hematuria) (9.2)		Palpable purpura (8.8)
	Endonasal ulcers (8.9)	Peripheral ulcerative keratitis (8.6)	Rapidly progressive acute respiratory failure (6.5)	Ischemic cerebrovascular event in young or without risk factors (9.0)	Nephritic syndrome (8.6)		Ulcers (7.8)
	Chronic rhinosinusitis with nasal polyps (8.9)	Proptosis (7.9)		Acute-onset focal neurological deficits in young patients (8.4)			Nodules (7.6)
	Septal perforation (8.5)	Retinal vasculitis (7.7)		Subacute-onset focal neurological deficits (relapsing–remitting or progressive) (8.4)			Urticaria (6.0)
	Unexplained laryngo-tracheal stenosis with stridor (8.0)	Episcleritis (6.7)		Subacute-onset encephalopathy (non–inflammatory–demyelinating) (7.6)			
	Saddle-nose deformity (7.7)	Papilledema (6.6)		Signs of pituitary involvement (e.g., diabetes insipidus) (7.0)			
	Chronic bilateral serous otitis media with conductive hearing loss (7.1)	Uveitis (5.1)					
	Chronic rhinosinusitis (6.9)						
	Sensorineural hearing loss (6.6)						
	Labyrinthine disorders (6.4)						

### 3.1 Systemic manifestations

The panel considered systemic symptoms as potentially relevant early indicators of AAV, although their intrinsic non-specificity was acknowledged. Persistent musculoskeletal pain, fever, asthenia and weight loss received moderate relevance scores, reflecting their role as supportive, rather than standalone, red flags, particularly when occurring alongside manifestations from other organs.

### 3.2 Otorhinolaryngology

ENT specialists identified upper airway disease as one of the most informative early domains for AAV. Refractory epistaxis and chronic crusting hemorrhagic rhinitis emerged as the highest-scoring red flags, followed by endonasal ulcerations, chronic rhinosinusitis with nasal polyps, and septal perforation. Laryngo-tracheal stenosis associated with stridor was also considered highly relevant. Other findings such as chronic serous otitis media, sensorineural hearing loss, and labyrinthine dysfunction received intermediate scores, reflecting their possible but less specific association with vasculitic involvement.

### 3.3 Ophthalmology

Among ophthalmologic symptoms, ocular pain, rapid visual blurring, and diplopia were rated as the most relevant indicators. Among clinical-detected findings, scleritis and peripheral ulcerative keratitis were found to have the highest level of consensus, followed by proptosis and retinal vasculitis. Episcleritis and papilledema received moderate relevance, while uveitis was considered less specific within the AAV spectrum. Overall, ocular findings were recognized as potential early red flags, particularly when persistent or unexplained by alternative diagnoses.

### 3.4 Pulmonology

Pulmonary specialists attributed strong relevance to stridor, given its association with subglottic stenosis, and to episodes of hemoptysis or blood−tinged sputum, which may signal alveolar hemorrhage. Persistent asthma, especially when poorly responsive to therapy, was also considered relevant, in the context of suspected EGPA. Localized wheezing and rapidly progressive respiratory insufficiency received intermediate scores, reflecting their diagnostic value in specific clinical contexts.

### 3.5 Neurology

Overall, neurological manifestations in the absence of alternative explanations - particularly in young adults - showed some of the highest relevance scores as red flags for AAV. Specifically, subacute focal neurological deficits of the central nervous system (CNS) in young individuals, with relapsing or progressive course, or encephalopathies with subacute onset achieved high agreement. Similarly, although in CNS AAV large vessel involvement is rare, there was consensus regarding the relevance of transient ischemic attack (TIA) occurrence or stroke-like acute syndromes in young patients without cardiovascular risk factors. Mononeuritis multiplex or asymmetric polyneuropathy were also consistently considered major red flags of the disease, whereas drug-resistant new-onset headache and signs of pituitary involvement, including diabetes insipidus, received intermediate levels of consensus as potential early indicators of CNS involvement in AAV.

### 3.6 Nephrology, gastroenterology and dermatology

Renal involvement was uniformly regarded as a high-value red flag domain. Acute or chronic kidney impairment associated with an active urinary sediment, including proteinuria and microscopic hematuria, received one of the highest mean scores in the entire project. Nephritic-pattern presentations also ranked highly. Other features such as new-onset hypertension or peripheral oedema were considered less specific and therefore not included among the strongest red flags.

Gastrointestinal manifestations were less frequent but recognized as potential red flags when present. Post-prandial abdominal pain suggestive of mesenteric ischemia, and mucous-bloody diarrhea, both received moderate relevance scores, reflecting their limited specificity but potential usefulness when associated with multisystem involvement.

Cutaneous findings were commonly endorsed as early and accessible signs of possible AAV. Palpable purpura, ulcers, and nodules were the highest-ranking features, whereas urticaria, although frequently observed, was considered less specific. Dermatologic manifestations were noted to be particularly informative when occurring concurrently with abnormalities in other organ systems.

### 3.7 Laboratory and first-level instrumental investigations

The Scientific Committee defined a set of first-line laboratory and instrumental investigations to be performed whenever AAV is clinically suspected (**Table 2**). These tests were not prioritized but were proposed as an initial comprehensive panel. This reflects the multisystem nature of AAV and the need for early, broad screening that can be adapted according to the patient’s clinical presentation.

**Table 2:** Suggested first-line laboratory and instrumental investigations according to clinical suspicion.

Laboratory	Imaging/Instrumental
Complete blood count abnormalities (acute anemia, chronic inflammatory anemia, thrombocytosis, neutrophilic leukocytosis, eosinophilia)	Non-contrast brain CT including the paranasal sinuses
ANCA testing	Chest X-ray/Chest CT
Elevated inflammatory markers (ESR, CRP)	Spirometry with DLCO
Increased serum creatinine (acute renal impairment)	Abdominal and renal ultrasound
Proteinuria	Echocardiography
Hematuria	Electroneurography
Presence of granular and cellular casts; dysmorphic erythrocytes	
Altered serum protein electrophoresis and immunoglobulin fractions	
Troponin	

CBC, complete blood count; ESR, erythrocyte sedimentation rate; CRP, C-reactive protein; DLCO, diffusing capacity of the lungs for carbon monoxide; CT, computed tomography.

Regarding laboratory investigations, abnormalities in the complete blood count, elevated inflammatory markers, renal function impairment, and the presence of proteinuria or hematuria were considered fundamental elements of initial assessment. Attention was given to eosinophilia, which is typically associated with EGPA, as well as to abnormalities in serum protein electrophoresis, including hypoalbuminemia and hypogammaglobulinemia. Troponin may be useful in assessing clinical or subclinical cardiac involvement. The examination of fresh urine sediment, especially the detection of dysmorphic erythrocytes, was noted as an important early indicator of glomerular involvement. ANCA testing completes the recommended first-line laboratory panel.

For instrumental and imaging investigations, the Committee agreed on a unified group of first-level assessments to be performed in case of symptoms supporting the possible involvement of the relevant organ including echocardiography, electroneurography, abdominal and renal ultrasound, chest X-ray or Computed Tomography (CT), spirometry with Diffusing Capacity of the Lungs for Carbon Monoxide (DLCO) measurement, and cranial CT including the paranasal sinuses. These tests were selected for their ability to identify early multiorgan involvement and provide guidance for subsequent specialty-specific second-level investigations. These are detailed separately in the corresponding tables.

## 4 Discussion

AAV are rare, but potentially life-threatening autoimmune diseases characterized by necrotizing inflammation of small- to medium-sized vessels and by a highly heterogeneous pattern of organ involvement (1). Consequently, due to the low specificity of the clinical syndromes associated with AAV, this diagnosis should be considered after excluding more frequent diseases. However, given its severity, AAV must always be considered whenever even subtle red flags are observed. In addition, despite major therapeutic advances - including B-cell–targeted therapies and complement inhibition - the prognosis remains strongly influenced by the timeliness of diagnosis, particularly in the early, often oligosymptomatic stages (5). This study addresses this critical issue by developing a multispecialty consensus-based set of red flags aimed at facilitating earlier recognition of AAV across different clinical settings.

A key finding of this project is the uneven diagnostic salience of different organ systems in early AAV. Manifestations involving the upper airways, kidneys, peripheral and central nervous systems, lungs, and eyes consistently emerged as the most relevant indicators of possible vasculitis. This reflects the known predilection of AAV for these vascular districts and supports existing evidence that ENT disease, active urinary sediment, otherwise unexplained subacute focal neurological symptoms in young adults, alveolar hemorrhage, and inflammatory ocular involvement often precede or accompany systemic disease evolution (15–19). In contrast, systemic symptoms such as fever, asthenia, or weight loss were rated as moderately relevant, highlighting their limited specificity when isolated, but confirming their contextual value when considered alongside with organ-specific findings.

The strong relevance attributed to upper airway involvement underscores the central role of necrotic granulomatous inflammation in GPA. Refractory epistaxis, chronic crusting rhinitis, septal perforation, and subglottic or tracheal stenosis were recognized as particularly informative, in line with the concept that localized ENT disease may represent an early or even isolated manifestation of vasculitis before systemic dissemination (20). Recurrent nasal polyposis, along with an increase in blood eosinophils, may also represent signs of EGPA (1). Similarly, the high scores assigned to renal red flags, especially the combination of renal dysfunction with active urinary sediment, reinforce the pivotal role of pauci-immune necrotizing glomerulonephritis as both a diagnostic and prognostic hallmark of AAV (21).

Neurological manifestations also ranked among the most relevant red flags. The prominence of unexplained acute, subacute, or relapsing-remitting focal neurological deficits particularly in young adults, reflects the vulnerability of CNS vessels as well as of the *vasa nervorum* to small-vessel vasculitis and the frequent under-recognition of this pathogenic mechanism when the nervous system is involved (15).

Ophthalmologic involvement emerged as another high-impact domain, particularly when inflammatory conditions such as scleritis, peripheral ulcerative keratitis, or retinal vasculitis were present. These manifestations are well recognized as markers of systemic activity and are often associated with aggressive disease phenotypes (22). Their inclusion among the most relevant red flags emphasizes the need to consider an underlying vasculitis and to prompt systemic medical evaluation in patients presenting with unexplained inflammatory eye disease.

Pulmonary manifestations showed a variable but meaningful diagnostic weight. Stridor, reflecting subglottic involvement, and hemoptysis or blood-stained sputum, suggestive of alveolar hemorrhage, were rated as highly relevant. Persistent or atypical asthma was also considered informative, particularly in the context of EGPA, while acknowledging that respiratory symptoms may overlap with more common pulmonary disorders, contributing to diagnostic delay.

The laboratory and first-level instrumental investigations proposed in this study further reflect the pathophysiological core of AAV, rather than serving as confirmatory tools. The emphasis on inflammatory markers, renal parameters, urinary sediment, eosinophilia, serum protein abnormalities, and ANCA testing mirrors the systemic immune-mediated nature of these diseases (23). Importantly, these tests were conceived as part of an integrated screening approach rather than as isolated diagnostic criteria, consistent with current concepts that no single laboratory marker is sufficient to diagnose AAV in the absence of compatible clinical features.

As AAV frequently presents outside rheumatology or nephrology settings and diagnostic delay remains a major contributor to irreversible organ damage (5), the presence of one or more high-relevance red flags, particularly when involving multiple organ systems, should prompt consideration of an underlying vasculitic process, support early laboratory assessment including ANCA testing and lead to timely referral to appropriate specialists. The checklist is intended to serve as a clinical alert tool, raising suspicion and facilitating multidisciplinary evaluation, rather than functioning as a rigid diagnostic algorithm.

Several limitations should be acknowledged. The consensus-based design does not provide quantitative validation of the proposed red flags and does not allow estimation of diagnostic accuracy measures such as sensitivity or specificity. Prospective external validation is therefore required. Moreover, the inclusive approach adopted may favor sensitivity over specificity, potentially increasing false-positive suspicion, particularly because some systemic or organ-specific manifestations (e.g., constitutional symptoms or isolated respiratory findings) are relatively non-specific and may occur in a wide range of alternative conditions. However, in AAV, where diagnostic delay is associated with significant morbidity, this trade-off is consistent with the intended screening purpose of a red-flag strategy.

In conclusion, this study provides a vasculitis-centered, multispecialty framework for the early recognition of ANCA-associated vasculitis. By focusing on clinical-symptoms and organ-specific manifestations rooted in the pathophysiology of small-vessel inflammation, the proposed red flags have the potential to shorten diagnostic delay, facilitate timely referral, and ultimately improve outcomes in patients with AAV. Future prospective studies are needed to validate the diagnostic performance of the proposed checklist in real-world clinical settings, including assessment of sensitivity, specificity, and predictive values. Such studies should also evaluate its impact on time to diagnosis and explore its potential integration into clinical pathways or decision-support systems to enhance early recognition of AAV.

## Data Availability

The datasets generated and/or analysed during the current study are not publicly available but are available from the corresponding author upon reasonable request.
