# Supplementary material for: A multispecialty consensus-based red flag checklist for the early recognition of ANCA-associated vasculitis
Source: Front Immunol. 2026 Apr 23;17:1818414. doi: 10.3389/fimmu.2026.1818414 (PMC13149232; doi:10.3389/fimmu.2026.1818414)
Supplement: Supplementary file 1 [file Table1.docx]

# Supplementary Table 1. Initial red flags and diagnostic tests proposed by the Scientific Board and Champions

## A. Clinical Red Flags (by organ/system)

| Domain | Red Flags / Clinical Indicators |
| --- | --- |
| Systemic symptoms | Fever, asthenia, weight loss, anorexia, nausea, vomiting, persistent arthro-myalgias, chest pain, headache |
| Otorhinolaryngology (upper airways) | Epistaxis; septal perforation; saddle nose; chronic rhinosinusitis with/without nasal polyps; chronic crusting hemorrhagic rhinitis; endonasal ulcers; chronic bilateral serous otitis media with hearing loss; sensorineural hearing loss; unexplained laryngo-tracheal stenosis |
| Ophthalmology | Proptosis; “red eye”; uveitis; retinal vasculitis; scleritis; episcleritis; diplopia; papilledema; peripheral ulcerative keratitis; visual blurring; pain with ocular movements |
| Neurology | Acute focal neurological deficits in young individuals lasting hours or recurring; subacute focal deficits with relapsing-remitting or chronic-progressive course, non–inflammatory-demyelinating; subacute encephalopathies; recurrent severe headaches, especially drug-resistant |
| Pulmonology | Hemoptysis/hematoid sputum; acute or progressive dyspnea; persistent dry cough; stridor; wheezing; asthma |
| Nephrology | New-onset hypertension; peripheral edema; nephrotic syndrome; chronic kidney failure with active urinary sediment (proteinuria, microscopic hematuria) |
| Gastroenterology | Post-prandial abdominal pain; mucous-bloody diarrhea |
| Dermatology | Nodules; ulcers; purpura; urticaria; rash |

## B. Laboratory Tests Proposed for Suspected AAV

| Level | Laboratory Investigations |
| --- | --- |
| Level I | CBC abnormalities (acute anemia, chronic inflammatory anemia, thrombocytosis, eosinophilia, neutrophilic leukocytosis); elevated inflammatory markers (ESR, CRP, fibrinogen); elevated creatinine; proteinuria; microscopic or macroscopic hematuria; granular and cellular casts; dysmorphic RBCs; elevated CPK; troponin; abnormal serum protein electrophoresis (QPE) |
| Level II | ANCA testing (MPO/PR3-specific); complement levels; ANA testing |

## C. Imaging and Instrumental Tests

| Level | Imaging / Instrumental Investigations |
| --- | --- |
| Level I | Echocardiogram; electromyography; chest X-ray; spirometry; diffusion capacity testing (DLCO); CT scan of paranasal sinuses (without contrast) |
| Level II | Brain MRI (with T2 hyperintensity assessment); CT scan for organ damage evaluation; Doppler ultrasound of supra-aortic trunks; transcranial Doppler |
